# Signature profile of cyclooxygenase-independent F2 series prostaglandins in *C*. *elegans* and their role in sperm motility

**DOI:** 10.1038/s41598-019-48062-y

**Published:** 2019-08-13

**Authors:** Ekta Tiwary, Muhan Hu, Michael A. Miller, Jeevan K. Prasain

**Affiliations:** 1 Department of Pharmacology and Toxicology, University of Alabama at Birmingham, Birmingham, Al USA; 2 Department of Cell Development and Integrative Biology, University of Alabama at Birmingham, Birmingham, Al USA

**Keywords:** Fatty acids, Fatty acids

## Abstract

We previously discovered that *Caenorhabditis elegans* synthesizes Cox-independent F-series prostaglandins (PGs). To delineate the Cox-independent prostaglandin pathways and evaluate their role in sperm motility in *C*. *elegans*, we developed a novel biochemical method for the rapid production of F-series PGs using arachidonic acid as the substrate and worm lysate as source of enzyme(s). Among the four F2-series PGs produced in the reaction, three of them were identified as 8-isoPGF2α, 5iPF2 VI, and PGF2α based on their retention times and MS/MS spectral comparison with standards using LC-MS/MS. PG production was not markedly affected by specific antioxidants, or Cox, Lox, and Cyp inhibitors, suggesting that these PGs are formed through a novel, biologically regulated mechanism in *C*. *elegans*. This study also assessed the ability of 8-isoPGF2α, 5iPF2 VI, PGF2α, and a mixture containing these PGs in a 0.5/0.08/1 ratio that reflects their synthetic composition to modulate sperm motility in *fat-*2 mutants. PGF2α and the PG mixture at 25 μM concentration significantly stimulated sperm velocity by 28% and 38%, whereas 8-isoPGF2α and 5iPF2 VI reduced the velocity by 21% and 30%, respectively, compared to vehicle control. These results indicate that the sperm motility effects of PGs are structure- and composition-dependent in *C*. *elegans*.

## Introduction

Prostaglandins (PGs) are lipid signaling molecules derived from 20 carbon polyunsaturated fatty acids (PUFAs) such as arachidonic acid (AA), dihomo-gamma-linolenic acid (DGLA) and eicosapentanoic acid (EPA)^1^. They are known to impact many biological processes including development, reproduction, immunity, inflammatory diseases, and cancer^2,3^. Classically, these bioactive PGs are synthesized via the cyclooxygenase (Cox) enzymes, which convert AA to PGH2^1,4,5^. Various PG synthases further convert this PGH2 to PGD2, PGE2, and PGF2 in specific tissues^1^. The canonical Cox mediated pathway of PG synthesis has been extensively studied in many organisms. However, other non-Cox mediated pathways have also been reported, including both enzymatic and non-enzymatic mechanisms. For example, the coral *Plexaura homomalla* synthesizes 15R-PGs through a novel 15R-Cox isozymes^6–8^. The insect firebrat *Thermobia domestica* and tobacco hornworm *Manduca sexta* synthesize PG-like compounds via lipoxygenase (Lox) enzyme^9,10^. PGs derived from non-enzymatic mechanisms generate a broad spectrum of PGs (also known as isoprostanes) and are mediated through the free radical-induced peroxidation of PUFA precursors^11^. These PGs can be differentiated from their biosynthetic isomers based on their unique stereochemistries. In addition to the above pathways, alternative pathways for specific PG synthesis are also reported in lower organisms such as nematodes, arthropods and yeasts^12,13^.

The nematode, *C*. *elegans* synthesizes PGs via an uncharacterized, Cox-independent mechanism^14,15^. To date, no Cox-like enzyme has been identified in the *C*. *elegans* genome, yet these nematodes synthesize abundant levels of a specific class of F-series prostaglandins (PGFs)^15^. In *C*. *elegans*, these PGFs are synthesized in the oocytes and play a critical role in sperm chemotaxis and motility^14,16^. Previous results from our lab demonstrated that this Cox-independent pathway may be conserved in mammals. Cox-1/Cox-2 double knockout mice and human follicular fluid showed distinct PG profiles that match that of *C*. *elegans*^16,17^. Furthermore, these PG profiles differed significantly from that of Cox- and ROS-derived PGs. The identification of this novel Cox-independent pathway may have strong clinical implications as this pathway is refractory to NSAIDs, whose effect on PG levels play a major role in regulating many biological processes including fertility and cancer^18,19^.

In this study, an *in vitro* assay was developed to facilitate the identification of key players in this pathway and to provide further insights into this Cox-independent PG synthesis mechanism. We show that Cox-independent PGs could be produced *in vitro* with a small volume of worm lysate (WL) and have functional consequences for sperm motility in *C*. *elegans*.

## Results

### *In vitro* non-Cox mediated PG production

Previous studies from our lab demonstrated that *C*. *elegans* produces three F-class PGs despite the lack of Cox encoding genes in its genome^14,15^. These PGs, PGF1, PGF2, and PGF3, are derived from DGLA, AA, and EPA PUFA precursors, respectively. The mechanism for PG synthesis in *C*. *elegans* has yet to be identified. To facilitate the identification of the key components of this pathway, we first developed an *in vitro* assay. To this end, we initially examined whether WL can produce PGs when exogenous PUFAs are added to the reaction. Crude WL was incubated with or without 100 µM DGLA, AA, or EPA in Tris-Cl buffer (pH 8, 100 mM) for 10 min at room temperature. The reaction products were extracted and analyzed by LC-MS/MS operated in multiple reaction monitoring (MRM) mode. In MRM, mass transitions *m/z* 355/311, 353/193 and 351/193 are indicative of PGF1α, PGF2α, and PGF3α, respectively. When WL was incubated with DGLA or AA, MRM analysis of the reaction products showed significant peaks at retention time (Rt) 11.8 and 11.7 min (Supplemental Fig. 1D,E) that correspond to PGF1α (Rt 11.8 min) and PGF2α (Rt 11.7 min) standards (Supplemental Fig. 1A,B), respectively. However, incubation of WL with EPA did not yield detectable levels of PGF3α. This suggests that the WL is enzymatically active in this *in vitro* reaction and can convert exogenous DGLA and AA into its corresponding PGs (Supplemental Fig. 1F). We next examined whether endogenous PUFA levels in the WL would be sufficient to produce detectable amounts of PGs. LC-MRM analysis with the mass transition *m*/*z* 353/193 showed detectable levels of PGF2α in WL without the addition of exogenous AA, indicating that sufficient AA is present in the WL (Supplemental Fig. 1H). However, F1 and F3 class PGs were not detected in the WL without the addition of exogenous DGLA and EPA (Supplemental Fig. 1G,I).

Next, we hypothesized that the PGs synthesis is enzymatic under *in vitro* condition and denaturing the WL would abolish the conversion of AA to PGF2α. To test this, we boiled the WL before incubating with AA. MRM analysis of boiled WL, incubated with AA, showed approximately 79% reduction in PG production when compared to control (Supplemental Fig. 2), suggesting the PGs produced in the *in vitro* reaction is mediated by an enzymatic mechanism.

A long-term goal of this study is to use an *in vitro* approach to identify the enzyme(s) responsible for PG production. Understanding where this enzyme(s) is localized can provide insight into the identity of this protein(s). To identify the cellular compartment that is reponsible for the maximal PG synthesizing activity, we fractionated the WL into the soluble (cytosolic) and particulate (membrane) fractions by differential centrifugation. MRM chromatograms (mass transition *m*/*z* 353/193) showed that each of the subcellular fractions obtained after differential centrifugation can convert AA to PGF2α. However, the maximum PGF2α level was produced with the soluble fraction (Supplemental Fig. 3).

**Figure 1:**
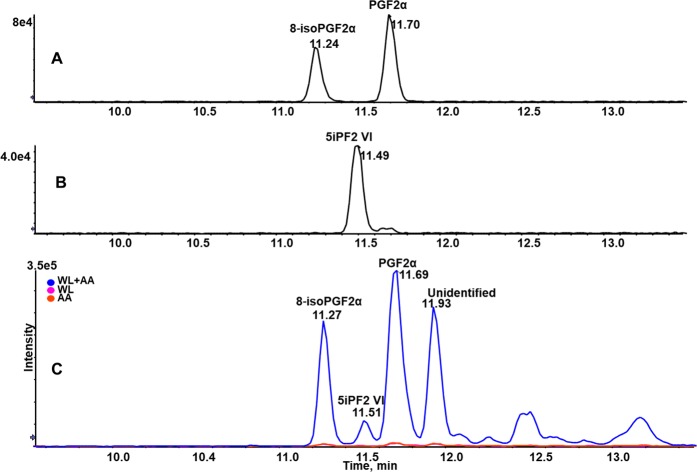
MRM chromatograms with the mass transition *m/z* 353/193 showing synthesis of four isomers of F2-series PGs after incubating AA with WL. Peaks eluted at Rt 11.2, 11.5 and 11.7 min corresponded to the standards 8-isoPGF2α, 5iPF2 VI and PGF2α, respectively; however, peak eluting at Rt 11.7 min remained unidentified. (**A**) 8-isoPGF2α, PGF2α standards (1 ng/ml) eluted at Rt 11.2 and 11.7 min, respectively. (**B**) 5iPF2 VI (10 ng/ml) eluted at Rt 11.4 min. (**C**) *In vitro* reaction product showing four major peaks eluted at Rt 11.2, 11.5, 11.7 and 11.9 min.

On reacting the soluble fraction with 100 µM AA for 10 min, four major peaks appeared at Rt 11.2, 11.5, 11.7 and 11.9 min in the MRM chromatograms (mass transition *m/z* 353/193) (Fig. 1). Among the four MRM peaks, Rt 11.2, 11.5 and 11.7 min corresponded to 8-isoPGF2α, 5iPF2 VI and PGF2α, respectively, based on matches with synthetic standards (Fig. 1A–C). We could not find a matching standard for the MRM peak eluting at Rt 11.9 min, so it remains unidentified. Each experiment was performed in triplicate along with controls (WL alone and AA alone). PGF2α-d_9_ was used as an internal standard to examine the extraction and ionization efficiency of the products. Together, these experiments showed that WL converted AA into four isomers of PGF2α via enzyme(s) and the soluble fraction exhibited major activity. Therefore, the soluble fraction was used for all subsequent experiments.

### Optimization

**Figure 2:**
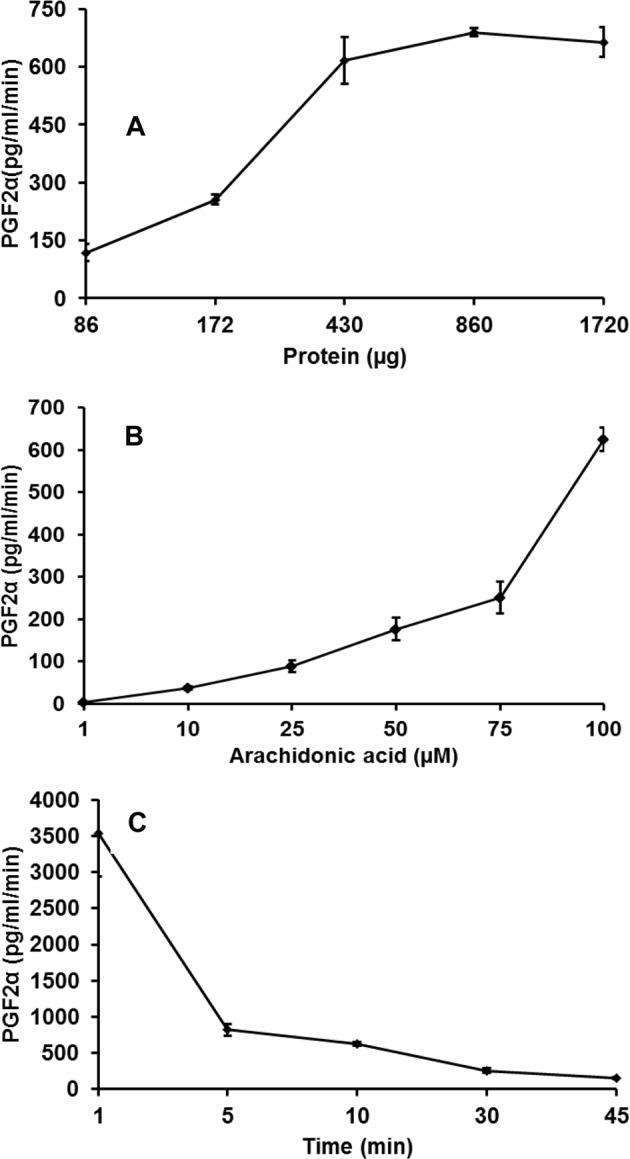
PGF2α synthesis is rapid and depends on WL and AA concentrations. (**A**) Different concentrations of WL (90–1700 µg protein) in reaction with 100 µM AA. (**B**) AA concentration range 1–100 µM in reaction with 450 µg WL soluble protein. (**C**) Incubation of 450 µg of WL soluble protein with AA for 0–45 min. All the experiments were done in triplicates and average was plotted. Error bars are SD (±).

To obtain better yield under *in vitro* conditions, we optimized the reaction conditions for PGF2α synthesis by altering the amounts of WL protein, AA and incubation time. PGF2α production increased with increasing concentrations of WL (Fig. 2A) and the reaction plateaued at a maximum yield of 690 ± 11.0 pg/ml/min obtained with 430 ± 20 μg of lysate protein. Further increase in WL protein did not increase the amount of PGF2α synthesized. Therefore, 430 ± 20 μg of lysate protein was selected for subsequent experiments with various concentrations of AA, over the range 1–100 μM (Fig. 2B). Increasing concentration of AA resulted higher levels of PGF2α and the maximum yield (626 ± 28.0 pg/ml/min) was obtained with 100 μM AA (Fig. 2B). PGF2α synthesis was found to be rapid, yielding 3,539 ± 595 pg/ml/min in the first min of incubation with AA (Fig. 2C); the rate was reduced to 822.3 ± 79.0 pg/ml/min after 5 min. We found that when the incubation time was increased to 30–45 min, the rate of PGF2α synthesis was reduced significantly. These experiments suggest that *in vitro* PGF2α synthesis is rapid and depends on protein and substrate availability.

### Characterization of PGs

Although MRM is considered a gold standard for quantitative analysis of known compounds, isobaric overlap can be a problem due to the low mass resolution of quadrupole mass analyzers. Therefore, we used high resolution and accurate mass measurement using a TripleTOF 5600 system, a hybrid quadrupole time-of-flight mass spectrometer (Qtof) to analyze the reaction products using LC-MS/MS. Furthermore, to ascertain that the added AA is the substrate that is converted in the *in vitro* reactions, ^13^C-AA (AA 1,2,3,4,5-^13^C) was incubated with WL and the products were analyzed. The molecular masses of ^13^C-AA derived PGF2s are 5 amu higher than that of its unlabeled counterparts. In the case of ^13^C PGF2α, the parent ion mass is *m/z* 358.249 and the major product ion mass is *m/z* 198.138.

**Figure 3:**
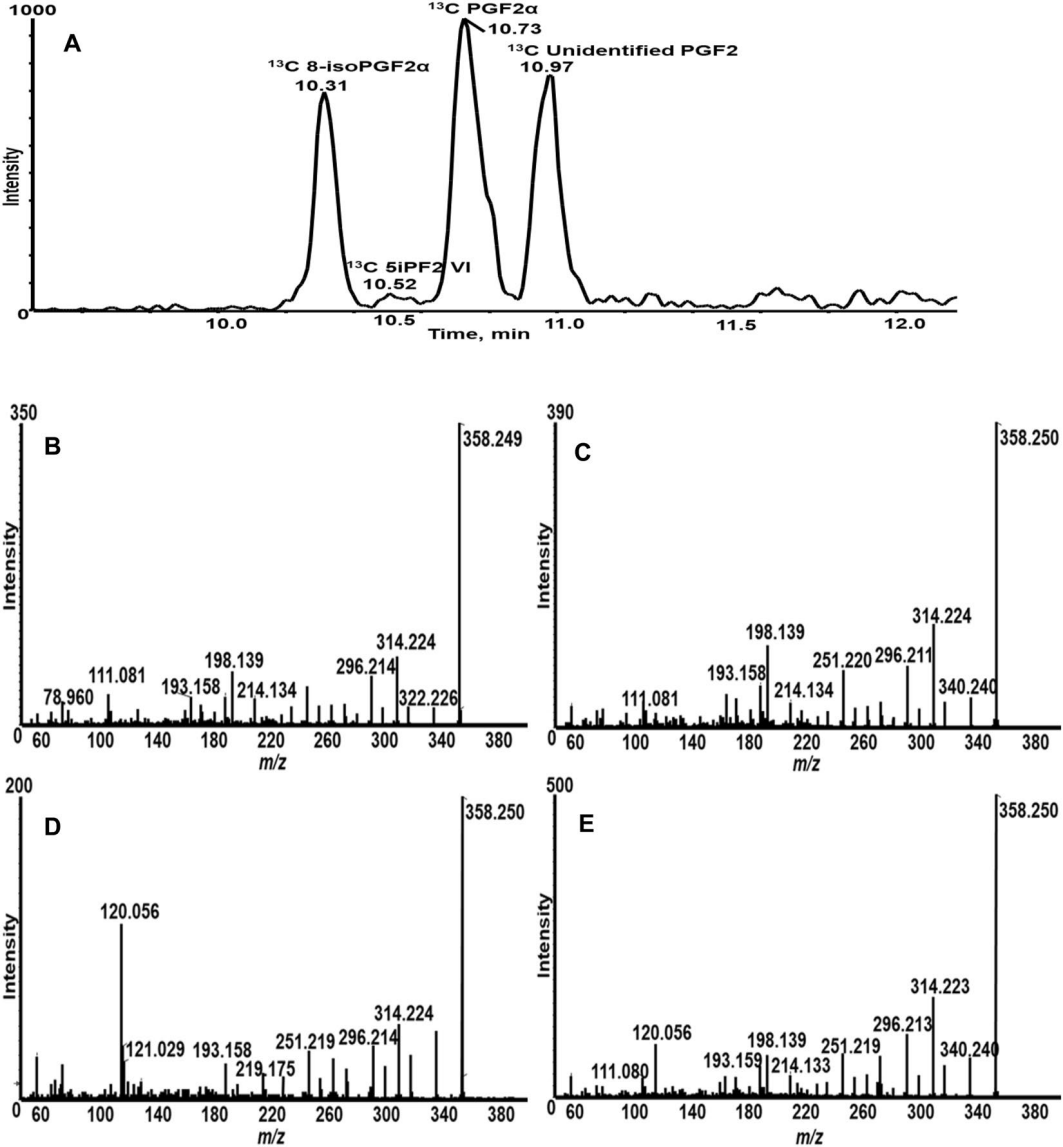
The accurate-mass precursor ion *m/z* 358.249 and its product ion *m/z* 198.139  showed  four iomers of ^13^C-F2-series PGs eluted at Rt 10.3, 10.5, 10.7 and 10.9 min  and fragmentation patterns of product ions allowed confirmation of the structures of three of them, namely, ^13^C-labeled 8-isoPGF2α, 5iPF2 VI and PGF2α. (**A**) Extracted ion chromatogram for *m/z* 358.249 and its production ion *m/z* 198.139.  MS/MS spectra  of* m/z* 358.249 at  Rt 10.3 min  (**B**);  Rt 10.7 min  (**C**);Rt 10.5 min  (**D**); and  Rt 10.9 min  (**E**).

An extracted ion chromatogram for *m/z* 358.249 (deprotonated precursor ion) and its product ion *m/z* 198.138 showed three major peaks eluting at Rt 10.3, 10.7 and 11.0 min and a minor peak at Rt 10.5 min in the reaction products of WL and ^13^C-AA (Fig. 3A). These peaks were not detected in the control (^13^C-AA alone). MS/MS spectra of the precursor ion *m/z* 358.249 at Rt 10.3 and 10.7 min showed major fragment ions such as *m/z* 198.140, 296.210, and 314.225 (Fig. 3B,C), which are 5 amu higher than those of the unlabeled counterparts as well as the major fragments from the 8-iso PGF2α and PGF2α standards. These analyses suggest that the peaks at Rt 10.3 and 10.7 correspond to 1,2,3,4,5-^13^C-8-isoPGF2α and 1,2,3,4,5-^13^C-PGF2α, respectively. The peak at Rt 10.5 min had a product ion base peak *m/z* 120.056 (cal. *m/z* 120.057 for C_5_H_8_O_3_^−^ 1,2,3,4,5-^13^C or *m/z* 115.040 from its unlabeled counterpart *m/z* 353.230) together with other product ions, such as *m/z* 296.211 and 314.224 (Fig. 3D). These ions are 5 amu higher than those of the unlabeled 5iPF2 VI standard, suggesting the peak at Rt 10.5 min is 1,2,3,4,5-^13^C-5iPF2 VI.

The LC-MS/MS peak *m/z* 358.249 eluting at Rt 10.9 min also generated the *m/z* 120.059 product ion, but with lower intensity than that of the Rt 10.5 min peak (Fig. 3E). Although the MS/MS spectra of this peak (Rt 10.9  min) showed many product ions that are characteristic of PGF2-like structures, we have yet to find a matching standard; therefore, the product remains unidentified.

### Effects of inhibitors on PG production

**Figure 4:**
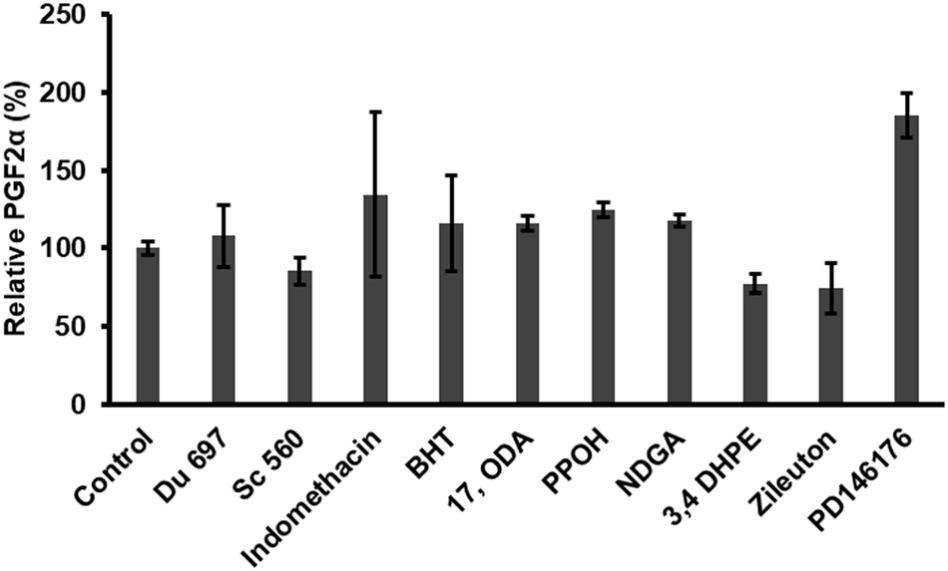
MRM-based quantification of PGF2α indicated that its levels were not markedly affected by antioxidants, Cox, Cyp and Lox inhibitors. Control reflects WL with AA, in the presence of DMSO (5.0% v/v) and PGF2α production in the reaction is considered as 100%. The values represent the average of three independent experiments. Error bars are SD (±).

We sought to identify the enzyme(s) potentially involved in PGF2α production in *C*. *elegans*. For this, we examined a number of inhibitors for their effects on PGF2α production. Antioxidants and Cox, Lox, and Cyp inhibitors were individually incubated with WL for 30 min prior to the addition of AA. Cox-specific inhibitors (DU-697, SC-560 and indomethacin) had no marked effects on PGF2α production, compared to the control (Fig. 4). Likewise, Cyp inhibitors 17-octadecynoic acid (17-ODA), and 2-(2-propynyloxy)-benzenehexanoic acid (PPOH) did not inhibit PGF2α production in our *in vitro* experiments. Specific Lox inhibitors (nordihydroguaiaretic acid (NDGA), 3,4 dihydroxyphenyl ethanol (3,4 DHPE), zileuton and PD146176) also had no effect on PGF2α production. To investigate whether PG production in *C*. *elegans* is mediated by free radicals, antioxidant (BHT, 100 µM) was incubated with WL. MRM analysis suggest that the addition of BHT had no effect on PGF2α synthesis. These data suggest that this non-Cox pathway in *C*. *elegans* is unique and not affected by any of the tested antioxidants or Cox, Lox, Cyp inhibitors.

### Differences between Cox- and Cox-independent pathways

One defining characteristic of the classical Cox pathway of synthesizing PGs is the production of a prostacyclin metabolite 6-keto PGF1α from the AA precursor. We were interested to know whether 6-keto PGF1α (*m/z* 369, deprotonated precursor ion) was synthesized in our *in vitro* experiments. To test this, AA was incubated with WL in the presence or absence of ovine Cox 1, and the reaction products were assessed by MRM. MRM chromatograms with the mass transition *m/z* 369/163 showed no detectable 6-keto PGF1α peak in the absence of Cox enzyme. This result coincides with our previous findings showing that 6-keto PGF1α is absent from whole worm lipid extracts^15^. However, a peak eluting at Rt 10.47 min, corresponding to 6-keto PGF1α standard, was detected when ovine Cox 1 was present in the reaction of WL with AA (Supplemental Fig. 4). Taken together, these results suggest that the enzyme(s) responsible for PG synthesis in *C*. *elegans* is characteristically different from the Cox proteins.

### Effect of PGs on sperm motility

**Figure 5:**
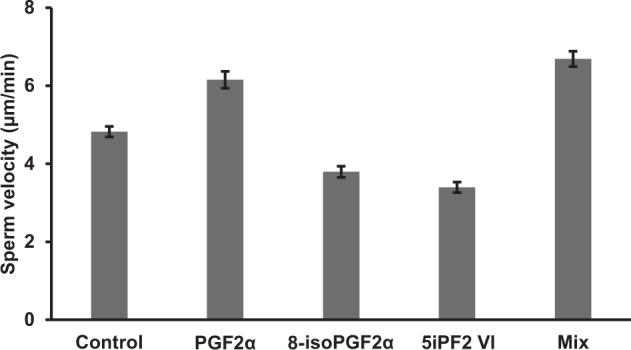
Sperm motility in the uterus of *fat 2* mutant hermaphrodites is modulated by PGs. *fat-2*(*wa17*) mutant hermaphrodites were mated with *fog-2*(*q71*) males. PGs were injected through the vulva of the mated hermaphrodites. Sperm in the uterus of *fat-2* mutant hermaphrodites are measured using time-lapse imaging. The numbers of sperm analyzed ranged between 86–121; means values are plotted and ±SEM are shown as error bars. *P* values (*P < 0.05) were calculated using a one-tailed student’s t test for PGF2α, and two-tailed test for 8-isoPGF2α, 5iPF2 VI and the PG mixture.

Previously our group has reported that PGF2α can alter sperm velocity^14,20^. In the current study we established that *C*. *elegans* synthesize 8-isoPGF2α, 5iPF2 VI and PGF2α; however, it is unknown whether 8-isoPGF2α and 5iPF2 VI also modulate sperm motility. To test this, we utilized *fat-*2 mutant worms that lack PUFA synthesis. These animals have low AA levels and well as low PGF levels. Based on our earlier published protocol^14,16,21^, we first mated *fat-2* mutant hermaphrodites with MitoTracker CMXRos-stained males. We then injected 25 µM of each compound individually or as a mixture containing 8-isoPGF2α, 5iPF2 VI and PGF2α (ratio 0.5/0.08/1 w/w, based on MRM analysis) through the vulva of the hermaphrodite. Injected worms were then rapidly mounted for time-lapse microscopy to measure sperm motility. Injected PGs showed differential effects on sperm motility. PGF2α and the PG mixture showed significant (p < 0.001) stimulatory effects, increasing sperm velocity by 28% and 38%, respectively, whereas 8-isoPGF2α and 5iPF2 VI reduced sperm motility by 21% and 30%, respectively, compared to vehicle control (Fig. 5). These data showed that 8-iso PGF2α and 5iPF2 VI had a negative impact on sperm velocity when they are used individually at high concentrations. However, when these PGs were injected as a mixture with PGF2α, their inhibitory role is not observed.

## Discussion

*C*. *elegans* synthesizes specific F-series PGs via an unidentified enzymatic pathway that acts independently of the classical Cox enzymes. We previously reported that this novel pathway, which is refractory to NSAIDs, may be conserved in mammals, including humans^16,17^. Since PGs regulate many physiologic and pathologic processes in the human body, the unconventional PG synthesis pathway identified in *C*. *elegans* may have significant clinical implications^17^. In order to identify the key players that are responsible for the Cox-independent PG synthesis, we develped an *in vitro* assay that will facilitate further downstream applications, such as target protein identification.

We first established that whole WL can convert free AA to specific F-series PGs in the *in vitro* assay. The MRM profiles of the synthesized PG products were similar to that of endogenous PGs extracted from whole worms^16^, indicating that the protein(s) is behaving similarly *in vitro* and *in vivo*. To localize where the activity is most robust, we separated whole WL into the cytosolic/soluble and membrane/particulate fractions by differential centrifugation. Compared to other fractions, incubation of free AA with the cytosolic fraction yielded the most PGF2α and other specific PGF2 isomers. This suggests that the protein(s) in this Cox-independent PG synthesis pathway is predominantly cytosolic. Although Cox-mediated PG synthesis is predominantly membrane bound^5^, cytosolic activity has also been reported^22^. A possible limitation to this finding is that the fractionation protocol was not fully optimized to obtain completely pure subcellular fractions, so the activity may be partly contributed by contaminents from broken membranes or organelles. However, the robust activity from the cytosolic fraction, in comparison to the insoluble fractions, minimizes this possiblity and suggests that the Cox-independent PG synthesis activity is mainly distributed in the cytosolic fraction.

Next, we confirmed that the reaction is indeed driven by an enzymatic mechanism, as opposed to ROS-mediated free radical oxidation. To this end, we boiled the WL and incubated the boiled WL with AA. MRM analysis of the resulting products showed significantly reduced levels of PGs, compared to that of unboiled WL, indicating the presence of a denaturable, presumably, enzymatic pathway for PG production in *C*. *elegans*. Our optimization studies also supported this observation. Increasing WL proteins yielded increasing levels of PGF2α, reaching a plateau at the higher WL concentrations, which is a typical characteristics of enzymatic reaction. Furthermore, PG production was also increased with increasing substrate (AA) concentration and the reaction is rapid, producing significant amounts of PGF2α within one min consistent with the presence of active enzyme. Under *in vitro* conditions, the autooxidation process is very slow and needs initiators to get detectable levels of PGs^23^.

Furthermore, our MRM data showed four specific isomers in the reaction product when AA was incubated with WL. To confirm that these isomers are derived from the AA and to help distinguish contaminant products in the reaction, we used stable isotope labelled ^13^C-AA (AA 1,2,3,4,5-^13^C) as the substrate for the reaction. The resulting mass shifts by 5 units (*m/z* 353.230 to 358.249) in the PG products indicated that ^13^C-AA was readily metabolized into ^13^C-PGs. These specific products further suggest that these reactions are not a result of auto-oxidation. In ROS-mediated mechanisms, a nonspecific spectrum of PG isomers, or isoprostanes, is formed and esterified to phospholipids. ROS-mediated isoprostanes are composed of 5-, 12-, 8-, or 15-series regioisomers, depending on the carbon atom to which the side chain hydroxyl is attached^24,25^. The formation of nonspecific, esterified PG species in racemic mixtures is considered a hallmark of non-enzymatic PG synthesis. The presence of specific products generated from the WL suggests that a novel, Cox-independent biochemical pathway is likely to be involved in their synthesis. Of particular interest is the possibility that the isoprostanes 8-isoPGF2α and 5iPF2 VI can also be produced via an enzymatic pathway. Much of the conventional literature have reported the use of these isoprostanes as markers of oxidative stress^21,22^. However, our data suggest that these compounds may be enzymatically regulated and may have novel developmental functions and regulatory mechanisms.

This Cox-independent PG synthesis pathway shows PUFA (substrate) specificity. While DGLA appeared to be the substrate for PGF1α, EPA did not generate identifiable PGF3α, though various other isomers sharing the same mass but different retention times were detected (mass transition *m/z* 351/193). These results are consistent with our earlier finding using whole worm lipid extracta^15^. Taken together, our data support the idea that the PG synthesis pathway in *C*. *elegans* is enzyme-mediated and not a result of free radical oxidation.

Classically, PG-endoperoxide synthase (a.k.a. cyclooxygenase or Cox) mediated PG synthesis is responsible for the bulk of PG production in many animals. Cox acts on AA to generate the bicyclic intermediate PGH2, which is further reduced to PGF2α, and its analogs^4,26^. A hallmark of Cox-mediated PG synthesis is the production of the PGI2 metabolite 6-keto PGF1α^14,15^. However, an interesting feature we observe in our *in vitro* data is the absence of the 6-keto PGF1α metabolite, suggesting that the pathway functions independently of Cox.

Specificity in product formation is a key in any biochemical reactions. We previously demostrated that *C*. *elegans* specifically synthesizes three major F-series PGs independent of Cox enzymes^15^. Interestingly, in this *in vitro* study, three major and one minor peaks corresponding to various F2-PGs (PGF2α, 8-isoPGF2α, 5iPF2 VI) derived from AA were detected with a peak ratio of 1/0.5/0.08. Our previous studies in human ovarian follicular fluid (HFF) from IVF patients and a Cox 1/Cox 2 knockout mouse showed a similar PG profile to those in *C*. *elegans* in terms of chromatographic retention times and MS/MS spectra^16,17^. This suggests that this novel Cox-independent pathway may be conserved in higher animals.

Next, to investigate whether the Cox-independent *de novo* pathways for PG synthesis are affected by other enzymes that have been reported to have PG synthesis activity, we evaluated the effects of a number of inhibitors on the *C*. *elegans* PGF2α production. The Cox inhibitors, indomethacin, DUP-696, SC-560, had no effect on PGF2α production. These results corroborate our earlier finding that *C*. *elegans* PG synthesis is refractory to NSAIDs, such as indomethacin, acetylsalicylic acid, CAY-10404 (specific Cox-2 inhibitor), and SC-560 (specific Cox-1 inhibitor), even at concentrations 10–500 times higher than reported IC_50_ values^11^. Other than Cox, Lox and Cyp enymes have also been reported to metabolize PUFAs to eicosanoids^9,10,27^. Therefore, we also tested the effects of Lox and Cyp inhibitors on PGs production. Similar to Cox inhibitors, Lox inhibitors, namely NDGA, 3, 4 DHPE, zileuton and PD146176, and Cyp inhibitors, 17, ODA and PPOH, showed no effect on PG synthesis, excluding the potential contribution of Lox and Cyp enzymes to *C*. *elegans* PGs synthesis. To further rule out the possiblity of an ROS-mediated mechanism for PGF2α synthesis *in vitro*, we included the antioxidant BHT (100 μM) in the *in vitro* reaction and showed it had no marked effect on PG production. Overall, these results suggest that PGs in *C*. *elegans* are formed through a novel, biologically regulated mechanism.

We previously reported that the PGF2α in *C*. *elegans* is important for sperm motility and guiding sperm to the oocytes^14,20^. However, little is known about the roles of the other F2 isomers produced in *C*. *elegans*. In this study, we injected three PGs (8-isoPGF2α, 5iPF2 VI and PGF2α) individually and as a mixture containing all three PGs in a 0.5/0.08/1 ratio into the vulva of PUFA deficient *fat-2* mutants. Since *fat-2* mutants lack the ability to produce PGs, sperms move to the spermatheca, or fertilization site, with reduced velocity^14,16^. It is important to note that we didn’t test the unidentified PGF2, eluting after PGF2α, due to lack of matching standards.

The reduced sperm velocity in *fat*-*2* mutants was rescued by injections of exogenous PGF2α and the PG mixture. However, injections of 8-isoPGF2α and 5iPF2 VI reduced the sperm velocity, compared to vehicle control. While the underlying mechanisms by which PGs influence sperm motility in *C*. *elegans* are unclear, the increased stimulatory effect of PGF2α in combination with the other PGs suggests that the combination of these compounds seems to have synergistic effects. A small caveat to the experiment is the backflow of injected PGs. While the injected concentration is 25 μM, some of the PGs may be lost through the vulva, rendering the working concentration to be less than the injected amounts.

Although the *C*. *elegans* genome does not encode Cox enzymes, a wide range of proteins with homology to mammalian PG synthases, PG transporters, PG reductases, cytochrome P450s, and phospholipases are present in its genome. Developing a reliable and reproducible assay for target identification is an important step. Our long-term aim is to identify the enzyme(s) required for Cox-independent PG metabolism and this study offers potential to help identify them using a small amount of lysate protein from *C*. *elegans*. Identifying the non-Cox enzyme(s) that catalyze PG metabolism may offer potential for discovering new PG functions in humans.

## Material and Methods

### Materials

The arachidonic acid (AA), eicosapentaenoic acid (EPA), dihomo-gamma-linoleic acid (DGLA) and PGs standards were purchased from Cayman Chemical Co. (Ann Arbor, MI, USA). 5-bromo-2-(4-fluorophenyl)-3-(4-(methylsulfonyl)phenyl)-thiophene (DUP-697), 5-(4-chlorophenyl)-1-(4-methoxyphenyl)-3-(trifluoromethyl)-1H-pyrazole  (SC-560), 17-octadeynoic acid (17, ODA), 2-(2-propynyloxy)-benzenehexanoic acid (PPOH), nordihydroguaiaretic acid (NDGA), 3,4-dihydroxyphenyl ethanol (3,4  DHPE), zileuton, 6,11-dihydro-[1]benzothiopyrano[4,3-b]indole  (PD146176) were also purchased from Cayman Chemical Co. (Ann Arbor, MI, USA). Indomethacin, taurine and butylated hydroxy toluene (BHT) were purchased from Sigma Chemical Co. (St. Louis, MO). All HPLC solvents and reagents were purchased from Fisher Scientific Co. (Norcross, GA) and were of HPLC grade.

### Worm cultivation and preparation of worm lysate

*C*. *elegans* wild-type N2 worms were maintained at 20 °C on nematode growth medium (NGM) plates. and fed with NA22 *E*. *coli*. For worm lysate, worms were seeded to 150 mm plates and incubated at 25 °C for 7 days. Concentrated bacteria (NA22 *E*. *coli*) were fed to the worms to prevent starvation. Worms were washed off the plates with M9 buffer and 2.5 g worms were aliquotted to polypropylene tubes and stored in −80 °C for lysate preparation.

For the preparation of worm lysate, 1 ml of 0.5 mm zirconium oxide beads and 1 ml of Tris-Cl buffer (100 mM), supplemented with protease inhibitors (Roche Applied Sciences, Germany) were added to 2.5 g of the frozen worms. The mixture was homogenized at speed 9 for 6–8 min using a bullet blender 5 homogenizer (Next Advance Inc., NY, USA). The homogenate was transferred to a 1.5 ml centrifuge tube and centrifuged at 16,000 × g for 10 min at 4 °C. The supernatant was transferred in new centrifuge tube stored at −80 °C until use. Protein concentration of the worm lysate was determined by BCA assay kit (Pierce™ BCA Protein Assay Kit, Thermofisher Scientific Inc. Norcross, GA, USA) following the manufacturer’s protocol.

### *In vitro* assay development and PGs extraction

For the synthesis of PGF2α and its isomers under *in vitro* conditions, 1 ml of reaction mixture was prepared with 100 mM Tris-Cl buffer (pH 8), 50 µl of 2 mM arachidonic acid (AA) and 50 µl of worm lysate (about 450 µg protein) unless otherwise stated. To determine the substrate specificity, other substrates (DGLA or EPA) were used, instead of AA, and a working 2 mM stock was prepared using 100 µl KOH (0.05%w/v), 100 µl substrate (20 mM stock in methanol) and 800 µl of ultrapure water.

The reaction mixture was incubated at room temperature for 10 min (unless otherwise indicated) and spiked with 10 µl of internal standard (10 ng/ml PGF2α-d_9_). Next, 4 ml of acidified (0.1% formic acid) methanol was added to the reaction mixture and the reaction was incubated on ice for 10 min to stop the reaction and inactivate the protein. The reaction mixture was centrifuged at 4,000 × g for 10 min at 4 °C, and the supernatant was collected and dried under nitrogen.

For the initial standardization, 50 µl of heme and 50 µl of 10-acetyl-3,7-dihydroxyphenoxazine (ADHP) were added to the reaction to check the requirement of cofactor for the reaction. Results showed that addition of cofactors did not increase PGF2α levels in the reaction and therefore the reactions were performed without adding heme and ADHP to the reaction.

### Analysis of PG products

Quantitative analysis of PG products was carried out by liquid chromatography tandem mass spectrometry (LC-MS/MS) using a Prominence 20A HPLC (Shimadzu, Kyoto, Japan) and 6500 Qtrap (Sciex, Framingham, MA) operated in multiple reaction monitoring (MRM) mode. Seperation of analytes was carried out by a gradient over a Synergi Hydro-RP column (Phenomenex, Torrance, CA) at 50 °C using mobile phase A 0.1% formic acid (FA) and B acetonitrile with 0.1% FA with a flow rate of 0.2 ml/min. The gradient started with 10% B and was increased to 80% at 11 min and become 100% B at 14 min. After that, mobile phase B was decreased to 10% at 16 min and allowed to re-equilibrate until 20 min total time had elapsed. Sample injection volume was 20 µl in all analyses. Column effluent was directed to the MS which was operating in negative mode. MS parameters were as follows: IS 4500 V, TEM 600, GS1 40, GS2 60, Curtain 20, and CAD 20. Compound parameters were as follows: Declustering potential (DP), collision energy (CE), and cell exit potential (CXP) are set at −80 V, −35 V and −11 V for F series PGs. In case of 5iPF2 VI, CE was −30. For MRM experiments, the mass transitions *m/z* 353/193 (PGF2α and its isomers), 351/191 (PGF3α and its isomers), 355/311 (PGF1α and its isomers), (PGF2α-d_9_, internal standard) and 369/163 (6-keto PGF1α) were used. For quantification of PGF2α, a series of working PGF2α solutions (0.001–20 ng/ml) containing 1 ng/ml internal standard PGF2α-d_9_ was prepared and analyzed by MRM. The standard curve showed excellent linearity in the 0.01–10 ng/mL range with a correlation coefficient >0.99. Post-acquisition data analysis was performed in Analyst v3.0.1 (Sciex) and PeakView v2.2 (Sciex).

### Characterization of ^13^C-labelled PG products

Analysis of reaction products with high mass accuracy of precursor and product ions was carried out by LC-MS/MS using a Prominence 20A HPLC (Shimadzu, Kyoto, Japan) and 5600 TripleTOF mass spectrometer (Sciex, Framingham, MA). Analyte separations were carried out by a gradient over a Synergi Hydro-RP column (Phenomenex, Torrance, CA) at 50 °C using a flow rate of 0.25 ml/min. Mobile phase A was 0.1% formic acid (FA) and B was acetonitrile 0.1% FA. Gradient starting conditions began at 10% B and was increased to 80% B at 11 min, increased to 100% B at 14 min, decreased to 10% B at 16 min and allow to re-equilibrate until 20 min total elapsed time. Sample injection volume was 20 µl in all analyses.

Column effluent was directed to the MS which was operated in negative mode ESI. MS parameters were as follows: IS 4500 V, TEM 400, GS1 50, GS2 50, and curtain gas of 25 psi. A precursor ion TOF MS Scan from 200–800 *m/z* for 250 ms and twenty 50 ms product ion scans from 50–1000 *m/z* were utilized to capture MSMS spectra. Product ion scans were set to a static 35 V CE with a 15 V CE spread for dissociation. Post-acquisition data analysis was carried out using Analyst 1.7 TF (Sciex) and PeakView v2.2 (Sciex).

### Differential centrifugation

Differential centrifugation was performed at 4 °C using an ultracentrifuge as shown in Supplemental Fig. 5. Briefly, homogenized WL was centrifuged at relatively low speed (1,300 × g for 5 min). The resulting supernatant 1, after centrifuging at 17,000 × g resulted in supernatant 2 and pellet C. Supernatant 2 was centrifuged at 80,000 × g for 60 min to obtain supernatant 3 and pellet D. Subsequent centrifugation of supernatant 3 at 150,000 × g for 3 h resulted in pellet E and supernatant (S).

### Inhibitor assay

Cox inhibitors (indomethacin, Sc560, DU 697), CYP inhibitors (17, ODA, PPOH, and lipoxygenase inhibitors (NDGA, 3,4 DHPE, zileuton, and PD146176) were prepared in DMSO and added to WL 30 min prior to the addition of substrate AA with a final concentration of 5.0% (v/v) DMSO. The antioxidant butylated hydroxy toluene (BHT 10 µM) was added to test for its effect on the inhibition of free radical-initiated PG products.

### Sperm motility assay

The effects of PGs on sperm speed was studied following the published protocol with minor modifications^14,16,21^. Briefly, 50–60 *fog-2*(*q71*) adult males were picked and transferred to new plate with a spot of bacteria containing Mitotracker CMXROS dye and incubated overnight at 16 °C to stain the males. Experiments were perfomed following our published protocol^21^. After 30 min of mating, hermaphrodites were mounted to a microscopic slide and 25 µM of 8-isoPGF2α, 5iPF2 VI or PGF2α were injected individually or as a mixture contaning these PGs in a 0.5/0.08/1 ratio into the vulva of the mated hermaphrodites. Time-lapse images were taken at 15 sec intervals for 5 min and sperm migration distance was measured using the NIS-Elements AR analysis 5.10.01 software. For each compound, experiments were done twice with three rounds of injections each time. Eight worms were injected per round of injection and images were captured from three worms using time-lapse imaging. For the final data, worms were selected from both batches and the average sperm speed and standard error of mean were calculated from 3–4 worms with a total of 86–121 sperm per expeirmental condition. Two-tailed Student’s t-test was performed for 8-isoPGF2α, 5iPF2 VI and the PG mixture. One-tailed Student’s t-test was used for PGF2α; p < 0.05 was considered significant.

## Conclusion

Our results suggest that Cox-independent PGs, including 8-isoPGF2α are formed with a signature profile through a biologically regulated mechanism. These PGs are biologically active and exhibit differential sperm motility effects in *C*. *elegans*. 8-isoPGF2α and 5iPF2 VI had inhibitory effects, whereas PGF2α and the mixture containing 8-isoPGF2α, 5iPGF2 VI and PGF2α stimulated sperm motility. The *in vitro* reaction using WL and AA, PGs production, appears to be unaffected by antioxidants and Cox, Lox, and Cyp inhibitors. The reaction exhibits saturation kinetics and is heat-sensitive, consistent with an enzyme-mediated process. A long term goal is to use this new assay to identify enzymes and intermediates involved in this alternate PG synthesis pathway.

## Supplementary information

Supplementary Figures
