# Supplementary material for: Signature profile of cyclooxygenase-independent F2 series prostaglandins in C. elegans and their role in sperm motility
Source: Sci Rep. 2019 Aug 13;9:11750. doi: 10.1038/s41598-019-48062-y (PMC6692340; doi:10.1038/s41598-019-48062-y)
Supplement: Supplementary file 1 — Supplementary Figures [file 41598_2019_48062_MOESM1_ESM.pdf]

Supplemental information for:

Signature profile of cyclooxygenase-independent F2 series prostaglandins in *C. elegans* and their role in sperm motility

Ekta Tiwary[1], Muhan Hu[2], Michael A. Miller[2¶], Jeevan K. Prasain[1*]

[1]Department of Pharmacology and Toxicology, [2]Department of Cell Development and Integrative Biology, University of Alabama at Birmingham, Birmingham Al, USA

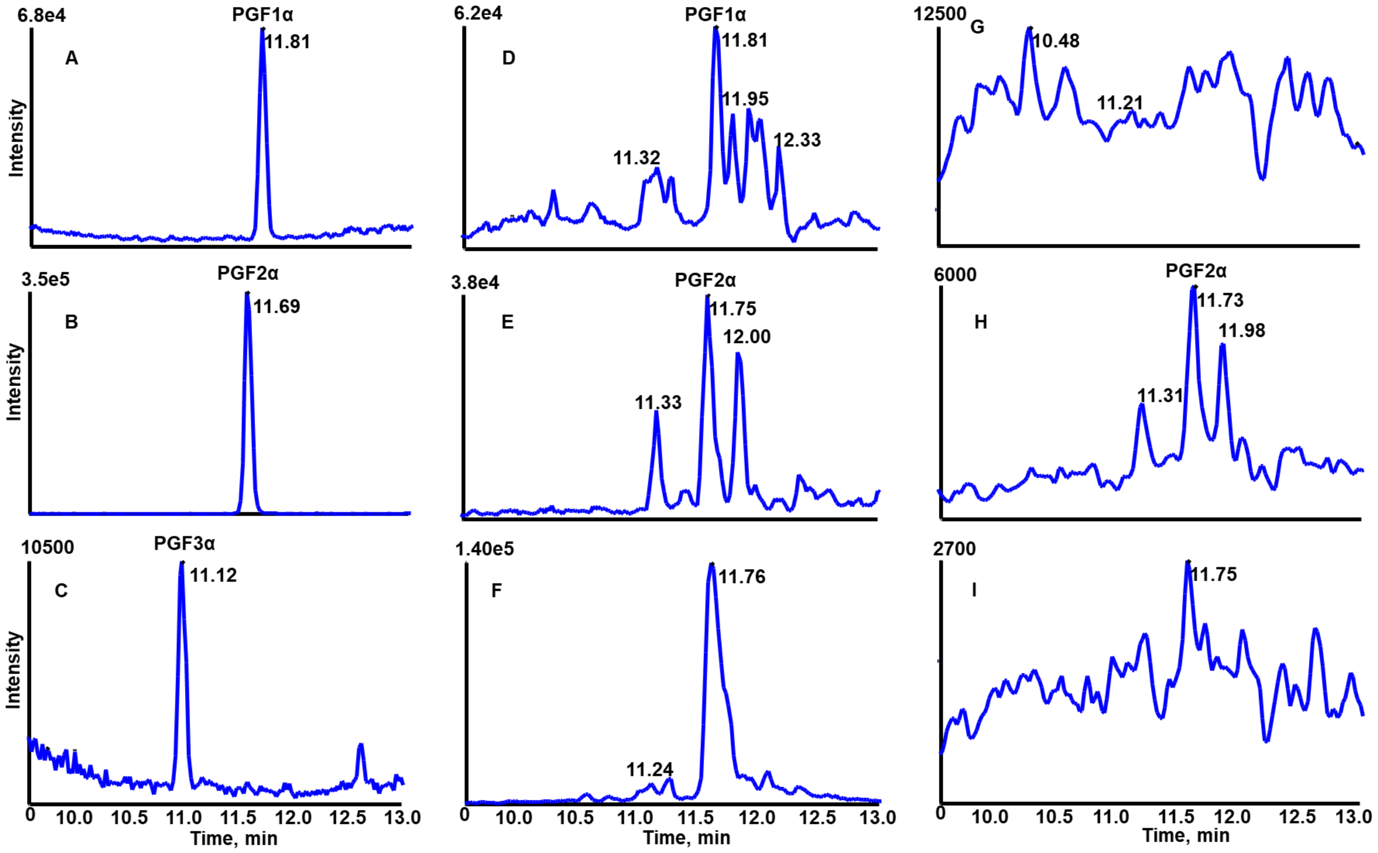

Supplemental Figure 1. MRM chromatograms with mass transitions *m/z* 355/311 and 353/193 showed that PGF1α (Rt 11.8 min) and PGF2α (Rt 11.7 min) are produced in WL reaction with DGLA and AA, respectively. F3 series PGs with mass transition *m/z* 351/193 were not matched with standards PGF3α (Rt 11.1 min) when WL was incubated with EPA.

A. PGF1α standard (1 ng/ml) with mass transition *m/z* 355/311. **B.** PGF2α standard (1 ng/ml) with mass transition *m/z* 353/193 **C.** PGF3α standard (1 ng/ml) with mass transition *m/z* 351/193 **D.** WL with DGLA. **E.** WL with AA **F.** WL with EPA **G-I** WL only with mass transitions *m/z* 355/311, 353/193 and 351/193, respectively.

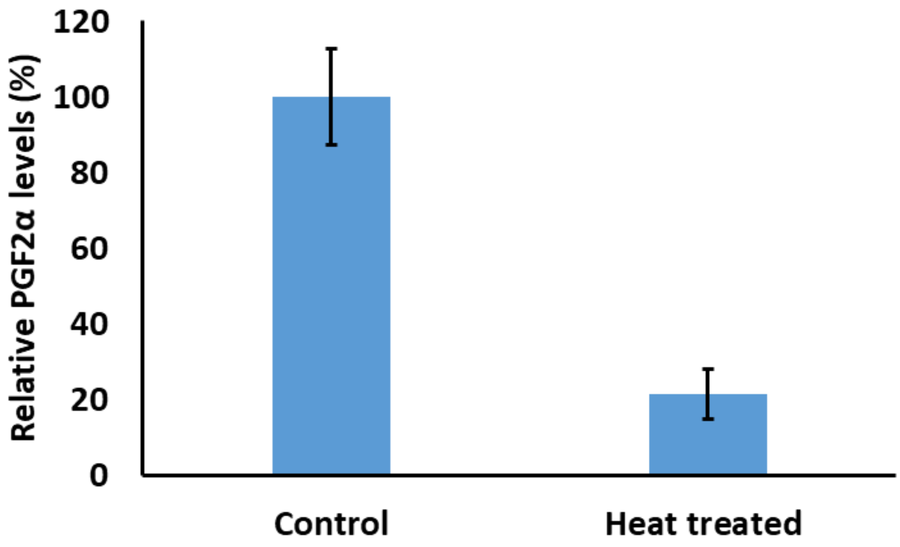

Supplemental Figure 2. MRM analysis of boiled and un-boiled WL showed that boiling of WL reduced PGF2α production. Error bars are SD (±)

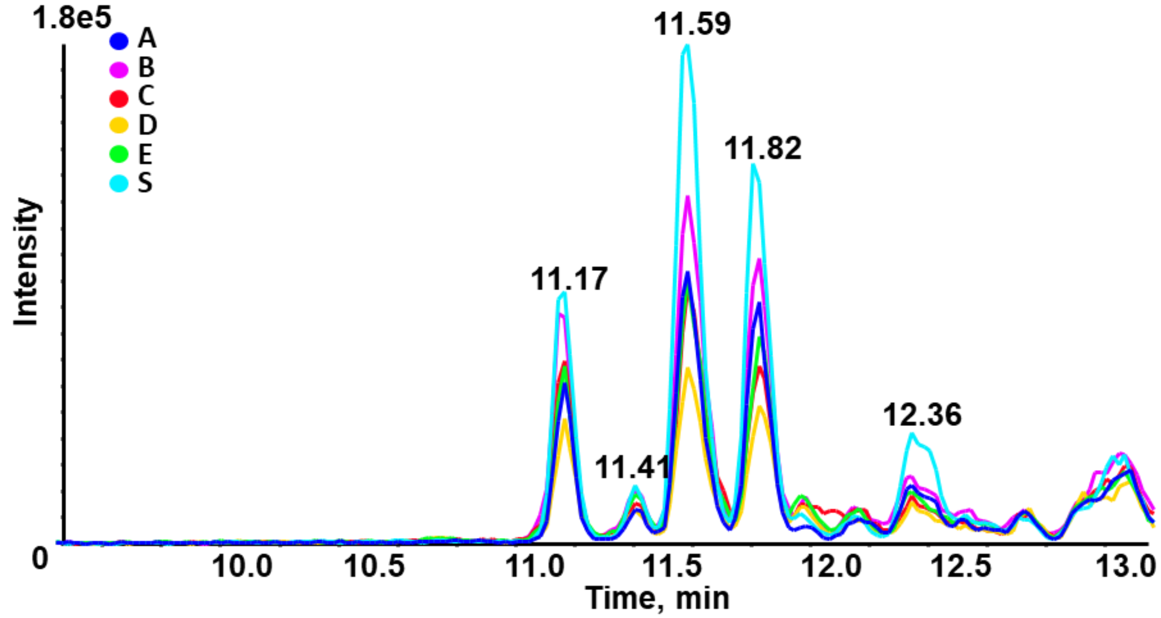

Supplemental Figure 3. PGF2α synthesis by insoluble and soluble fractions showed that soluble component had the highest activity compared to other fractions. Comparative MRM profiles of insoluble and soluble cellular components of WL (isolated as explained in method and Supplemental Fig. 5) with mass transition *m/z* 353/193, after addition of AA. Soluble fraction (S) showed maximum PGF2α synthesis (Rt 11.6 min)

A. whole WL **B.** Pellet obtained after 1300 X g rich in nuclei and debris **C.** Pellet obtained after 17,000 x g rich in mitochondria **D.** Pellet obtained after 80,000 x g rich in membrane **E.** Pellet obtained after 150,000 x g rich in ribosomes **S.** Soluble cytosolic fraction

A.

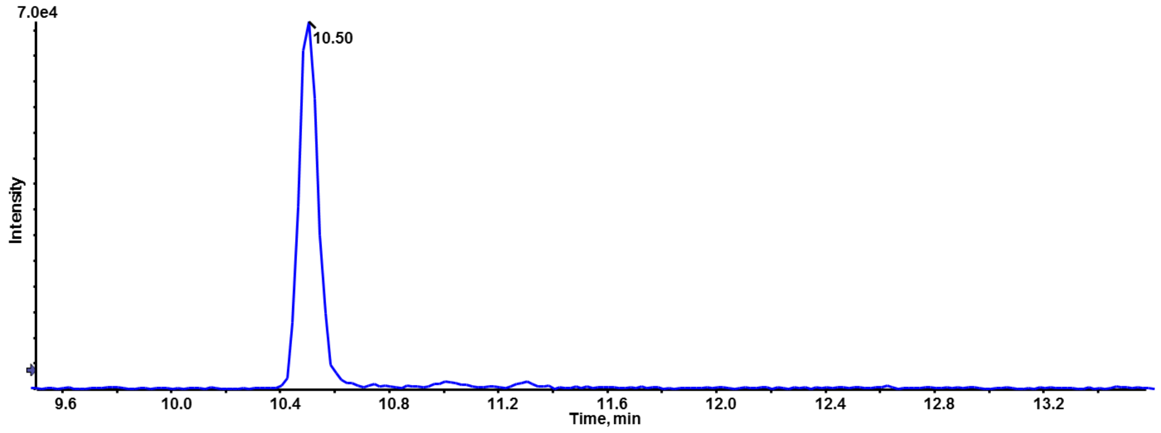

B.

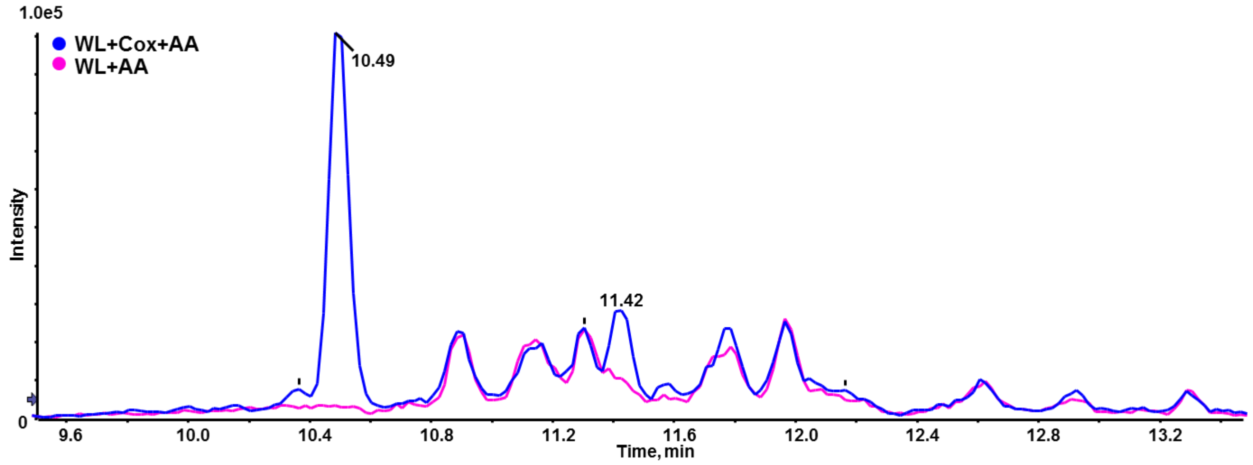

Supplemental Figure 4. MRM chromatogram with transition *m/z* 369/163 of WL with AA, in the presnece and absence of ovine Cox 1 showed that WL did not synthesize 6-keto PGF1α.

A. 6-keto PGF1α (1 ng/ml) standard Rt 10.5 min **B.** WL in the presence of ovine Cox 1 shows a major peak (Rt 10.49 min) corresponding to chemically synthesized 6-keto PGF1α standard.

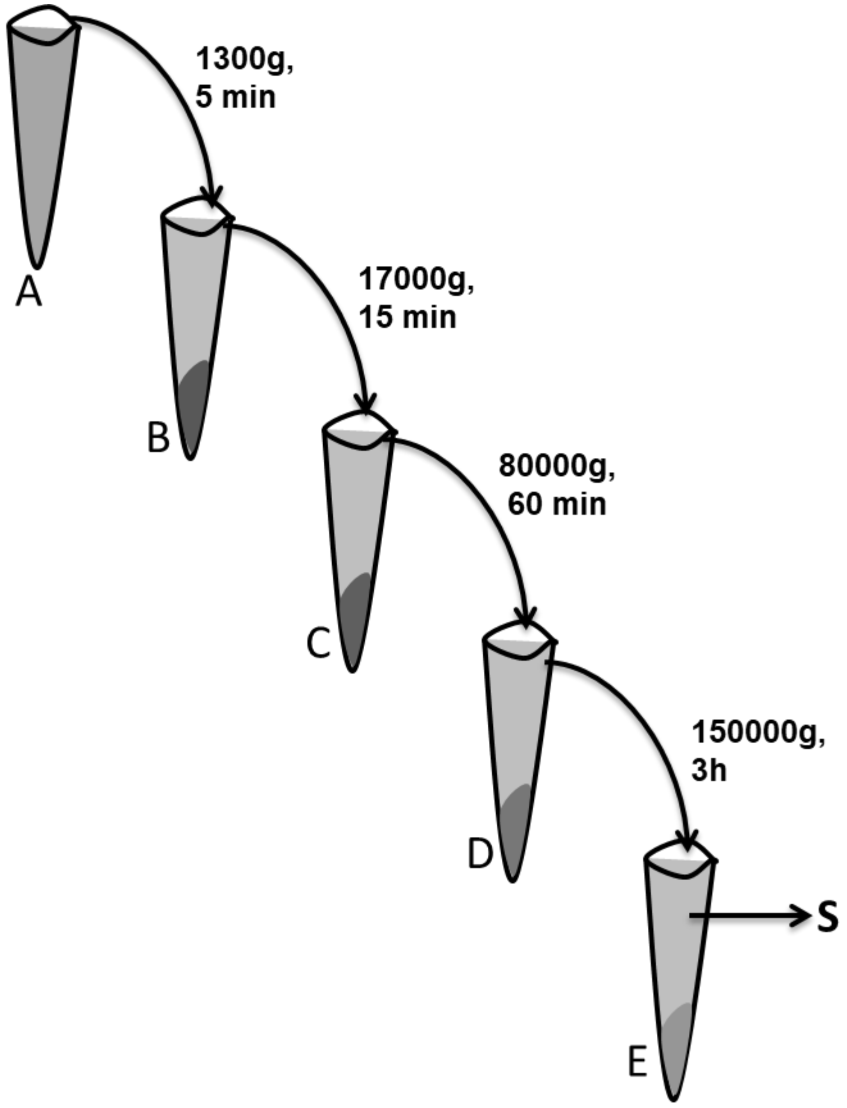

Supplemental Figure 5. Schematic representation of differential centrifugation of worm lysate
